# Cascaded deep learning for synthesis of ^18^F-FDG PET from ^18^F-octreotide: model parameter optimization and performance evaluation

**DOI:** 10.3389/fnume.2026.1920721

**Published:** 2026-10-16

**Authors:** Ruxi Chang, Liping Yang, Jungang Gao, Xiang Liu, Bo Wang, Yan Li, Xiaoyi Duan

**Affiliations:** PET/CT Center, The First Affiliated Hospital of Xi’an Jiaotong University, Xi'an, China

**Keywords:** 18F-FDG, 18F-octreotide, cascaded generative adversarial network, cross-tracer translation, deep learning, neuroendocrine tumors, PET image synthesis

## Abstract

**Objective:**

To investigate a cascaded conditional generative adversarial network (cGAN)–based method for synthesizing ^18^F-FDG PET images from ^18^F-octreotide, optimize model parameter configurations, and evaluate the quality of the synthesized images.

**Methods:**

Imaging data from 71 tumor patients who underwent dual-tracer PET/CT examinations with ^18^F-FDG and ^18^F-octreotide between May 2022 and November 2025 were retrospectively collected. A cascaded deep learning architecture (first stage: 3D U-Net; second stage: self-attention 3D V-Net) was employed to evaluate the effects of image preprocessing, resampling size, and patch size on synthesis quality. Three preprocessing strategies (log transformation, normalization, and a combined approach) were compared. Image quality was assessed using five objective metrics (SSIM, PSNR, MSE, MAE, and NMI) and Likert 5-point ratings from three nuclear medicine physicians; liver and lesion-level SUV agreement were further evaluated.

**Results:**

In the held-out test cohort (*n* = 14), the combined preprocessing method (log transformation + normalization) significantly outperformed the individual methods (SSIM: 0.8454 ± 0.0328 vs. 0.7831 ± 0.0337 vs. 0.7438 ± 0.0362, *P* < 0.001). On the identical held-out test cohort (*n* = 14), the Group 2 parameter configuration (resampling size: 256 × 256 × 512; patch size: 128 × 128 × 128) outperformed Group 1, with an SSIM of 0.8854 ± 0.0455 and a PSNR of 28.15 ± 3.35 dB, representing relative improvements of 19.1% and 83.5%, respectively, over Group 1 (paired-samples t-test, both *P* < 0.001). The mean Likert score was 4.53 ± 0.19. Quantitative analysis of liver SUVmean showed no significant difference between synthesized and real images (*P* = 0.967, paired t-test). Lesion-level quantitative analysis demonstrated high agreement between synthetic and real ^18^F-FDG images, with Pearson correlation coefficients of 1.0000, 0.9995, and 0.9991 for SUVmax and SUVmean (*n* = 14) and SUVpeak (*n* = 13), respectively (all *P* < 0.001), no significant differences on paired t-tests (all *P* > 0.05), and all Bland–Altman differences within the 95% limits of agreement.

**Conclusion:**

The cascaded deep learning model enabled cross-tracer synthesis from ^18^F-octreotide to ^18^F-FDG PET images. In this exploratory, single-center pilot cohort, the optimized configuration demonstrated promising quantitative and visual performance, supporting image-synthesis feasibility and potential clinical utility of a single-injection, dual-parameter molecular imaging approach for neuroendocrine tumors. Prospective multicenter validation is warranted before any clinical application.

## Introduction

Positron emission tomography (PET) is a sophisticated molecular imaging modality with indispensable applications in cancer diagnosis, staging, therapeutic monitoring, and prognostic evaluation. ^18^F-fluorodeoxyglucose (^18^F-FDG), currently the most commonly used PET tracer, enables the detection of malignancies by assessing tissue glucose metabolism. Nevertheless, ^18^F-FDG PET has several intrinsic limitations: certain tumors exhibit low metabolic activity and consequently low FDG uptake, which may reduce detection rates; moreover, inflammatory and infectious processes can also show increased FDG uptake, resulting in false-positive results (1).

Neuroendocrine tumors (NETs) are a heterogeneous group of neoplasms arising from neuroendocrine cells and can occur in multiple organs throughout the body, most commonly in the gastroenteropancreatic system and lungs. NETs exhibit marked biological heterogeneity, ranging from indolent growth to highly aggressive behavior, and accurate molecular classification is essential for treatment selection and prognostic evaluation (2). Somatostatin receptor (SSTR) imaging is a cornerstone for the diagnosis of NETs. As an SSTR agonist, ^18^F-octreotide (^18^F-OCT) binds with high affinity to overexpressed SSTR2 on tumor cell surfaces, enabling precise localization of both primary and metastatic lesions (3).

Nevertheless, clinical evidence suggests that assessment based solely on SSTR expression is inadequate. Approximately 15%–30% of NET patients exhibit SSTR–FDG discordance: SSTR-positive/FDG-negative patterns indicate well-differentiated, indolent, low-grade NETs that are more likely to benefit from peptide receptor radionuclide therapy (PRRT); conversely, SSTR-negative/FDG-positive findings reflect tumor dedifferentiation, increased proliferative activity, and poor prognosis, for which chemotherapy or targeted therapy should be considered; dual-positive lesions indicate heterogeneous tumor biology and require integrated evaluation (4, 5). Thus, simultaneous characterization of SSTR and FDG molecular features is critical for accurate staging and risk stratification of NETs.

Conventional dual-tracer imaging strategies require patients to undergo two separate PET/CT scans with ^18^F-octreotide and ^18^F-FDG on different days, leading to several drawbacks: (1) cumulative radiation exposure from two examinations (a previous whole-body ^18^F-FDG PET/CT dosimetry study reported an effective dose of approximately 14 mSv per examination); (2) an interval of several days to avoid tracer interference, prolonging the diagnostic workflow; (3) increased healthcare costs; and (4) a higher risk of patient attrition because of multiple appointments (6, 7). Therefore, the development of an AI-based approach to synthesize ^18^F-FDG images from a single ^18^F-octreotide PET scan may support a single-injection, dual-parameter molecular imaging paradigm.

In recent years, deep learning has made significant advances in medical image synthesis (8). Generative adversarial networks (GANs) and their extensions—particularly conditional GANs (cGANs) and the Pix2Pix framework—have been widely applied to cross-modal medical imaging tasks. Bi et al. (9) pioneered the use of multi-channel GANs for PET image synthesis, demonstrating the feasibility of deep learning–based PET generation. Shen et al. (10) further demonstrated the feasibility of cross-tracer PET image synthesis from ^18^F-FDG to ^11^C-CFT for the diagnosis of Parkinson's disease, providing new directions for cross-tracer molecular imaging. However, the synthesis of ^18^F-FDG from ^18^F-octreotide represents a cross-biological tracer transformation and introduces more substantial challenges than conventional image enhancement. The two tracers are based on fundamentally different biological mechanisms (SSTR expression versus glucose metabolism), leading to intrinsic differences in lesion contrast generation, physiological background uptake, and SUV dynamic range.

Existing studies have shown that single-stage end-to-end networks face inherent limitations in cross-modal medical image synthesis. Wang et al. used a 3D cGAN to estimate full-dose PET from low-dose PET; although overall structural similarity was satisfactory, the generated images exhibited blurred details and over-smoothing in metabolically active regions (11). In the field of cross-tracer synthesis, Lan et al. employed a 3D self-attention conditional GAN (SC-GAN) to synthesize amyloid PET from multi-contrast MRI and found that synthesis errors were significantly increased in brain regions with high amyloid burden, and that the synthesized PET could not replace real PET for clinical diagnostic tasks (12). A review by Yi et al. further pointed out (8) that single-stage GANs couple cross-domain mapping and detail restoration within the same feature space, often adopting conservative strategies to minimize the overall loss function, resulting in insufficient reconstruction of regions with high specific uptake. These findings suggest that single-stage architectures struggle to balance anatomical fidelity and metabolic feature translation, and that cascaded or multi-stage strategies are needed to decouple mapping tasks at different scales. At present, direct evidence for cross-tracer synthesis (e.g., FDG→octreotide) remains limited (13), and the limitations of single-stage networks are largely inferred from cross-modal MRI-to-PET synthesis studies.

Cascaded deep learning models overcome the above limitations through a task-decoupling strategy, decomposing complex cross-tracer mapping into an ordered optimization process: the first stage focuses on anatomically constrained basic synthesis (addressing the presence problem by ensuring accurate organ localization and reasonable background levels), while the second stage emphasizes the refined reconstruction of FDG-specific metabolic features (addressing the accuracy problem by correcting SUV quantitative bias and restoring lesion contrast) (11). This progressive optimization effectively separates cross-domain mapping errors from detail reconstruction errors, allowing the second-stage network to recover high-frequency details based on residual information. Gong et al. (14) demonstrated that pretraining the network with simulated data followed by fine-tuning only the final layers using real patient data can better adapt to the noise characteristics of clinical data. Therefore, adopting a cascaded architecture is essential for overcoming the domain gap between ^18^F-octreotide and ^18^F-FDG and breaking the performance limitations of single-stage models.

This study aimed to develop a cascaded deep learning–based method for synthesizing ^18^F-FDG PET images from ^18^F-octreotide and to systematically investigate the effects of image preprocessing methods and key model parameters (resampling size and patch size) on synthesis quality. By optimizing these configurations, this study sought to obtain high-quality synthetic ^18^F-FDG images as a potentially cost-effective auxiliary imaging approach for NETs, with the potential to reduce repeat imaging burden and healthcare costs.

## Materials and methods

### Study cohort

The workflow of the present study is illustrated in Figure 1. This retrospective study included patients with tumors who underwent dual-tracer PET/CT imaging with ^18^F-FDG and ^18^F-octreotide at the Department of Nuclear Medicine of our hospital between May 2022 and November 2025. The inclusion criteria were: (1) pathologically confirmed or clinically diagnosed neuroendocrine tumors, or other tumors requiring dual-tracer assessment; (2) completion of both ^18^F-FDG and ^18^F-octreotide PET/CT scans within a 1-week interval; and (3) adequate image quality for diagnostic interpretation. The exclusion criteria were: (1) severe motion or metal artifacts affecting image quality; (2) incomplete clinical data; and (3) any disease-related treatment or invasive procedures performed between the two scans. A total of 71 unique patients were enrolled, and each patient contributed one paired ^18^F-FDG/^18^F-octreotide examination. The cohort included 42 males and 29 females, with a mean age of 56.6 ± 13.9 years (range: 28–81 years). No missing imaging or clinical data required for the enrolled analyses were present. The dataset was randomly split into training (*n* = 43, 60.6%), validation (*n* = 14, 19.7%), and testing (*n* = 14, 19.7%) cohorts in a 6:2:2 ratio. There were no statistically significant differences among the three groups in terms of age, sex, BMI, or tumor type distribution (*P* > 0.05). The study was approved by the institutional ethics committee (Approval No.: 2022-LSYZ-(J2)), and informed consent was waived due to the retrospective design.

**Figure 1:**
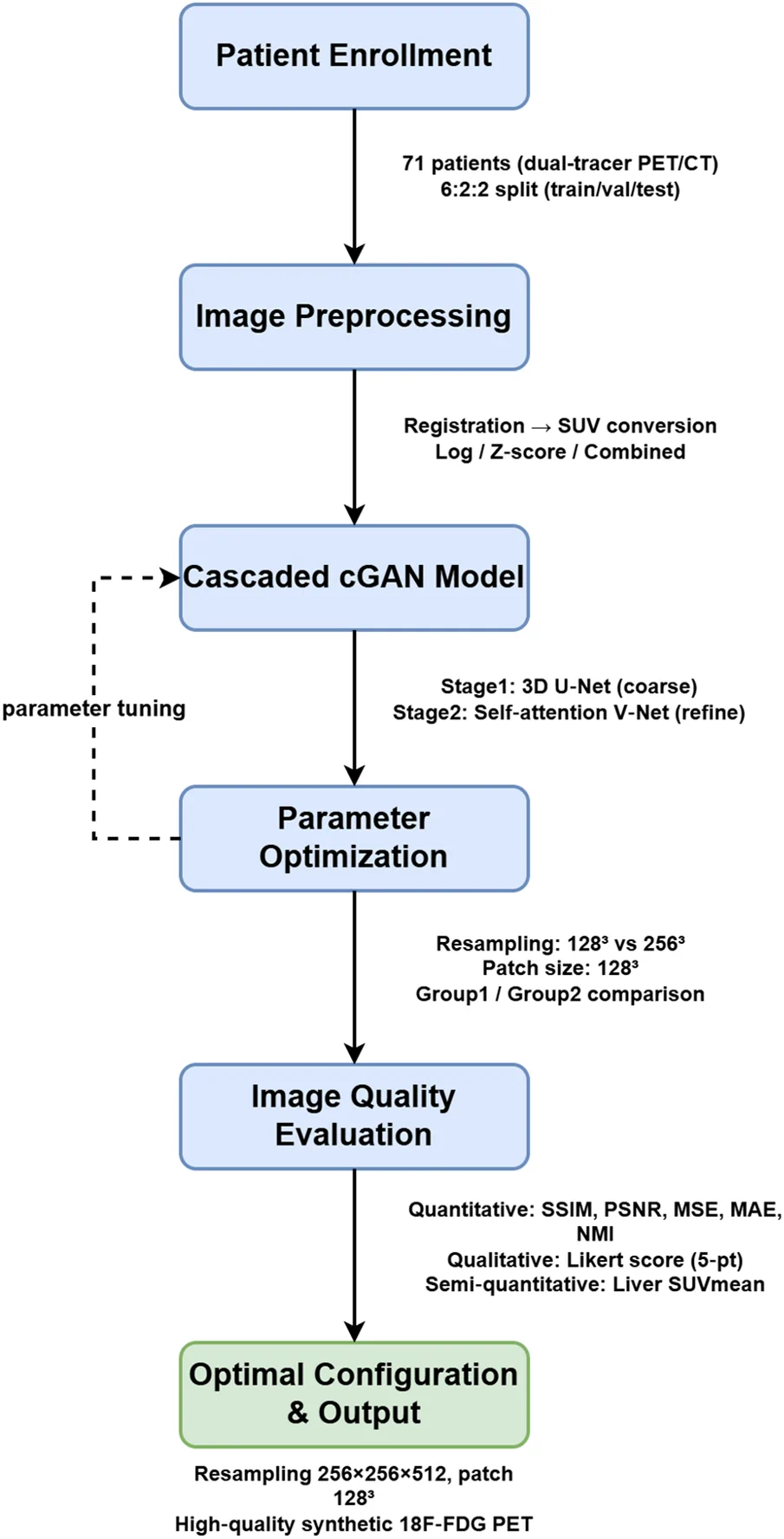
Workflow of the cascaded deep learning framework for synthesizing ^18^F-FDG PET images from ^18^F-octreotide PET data. The study enrolled patients with dual-tracer PET/CT examinations, split into training, validation, and testing cohorts. Images were preprocessed using three strategies (log transformation, Z-score normalization, and combined). The cascaded cGAN comprised a first-stage 3D U-Net for coarse synthesis and a second-stage self-attention V-Net 3D for refinement. Parameter optimization compared different resampling sizes with a fixed patch size, denoted as Group 1 and Group 2. Image quality was evaluated quantitatively (SSIM, PSNR, MSE, MAE, NMI), qualitatively (Likert 5-point scale by three physicians), and semi-quantitatively (liver SUVmean and lesion SUVmax, SUVmean, and SUVpeak). The optimal configuration generated high-quality synthetic ^18^F-FDG PET images.

### PET/CT acquisition and reconstruction

PET/CT imaging was performed using Philips Gemini TF 64 or Philips Vereos systems (Philips Healthcare, Best, The Netherlands). ^18^F-FDG was synthesized at the institutional PET center using an automated module (Tracerlab FX-FDG, GE Healthcare) with radiochemical purity exceeding 95%, while ^18^F-octreotide was prepared in the institutional radiopharmacy with purity exceeding 98%.

^18^F-FDG protocol: Patients fasted for at least 6 h, with fasting blood glucose maintained below 11.1 mmol/L. An intravenous dose of ^18^F-FDG (3.70–5.55 MBq/kg, 0.10–0.15 mCi/kg) was administered, followed by a 60-minute uptake period before whole-body PET/CT imaging. CT acquisition was performed at 120 kV with automatic tube current modulation (50–300 mA) and a pitch of 0.641. PET data were acquired in 3D mode for 2–3 min per bed position over 6–8 bed positions, covering the region from the skull base to mid-thigh.

^18^F-octreotide protocol: No specific preparation was required. Patients received an intravenous injection of ^18^F-octreotide at 2.96–3.70 MBq/kg (0.08–0.10 mCi/kg), followed by a 50–60-minute uptake period prior to whole-body PET/CT scanning. CT acquisition parameters were consistent with those used for ^18^F-FDG imaging. PET acquisition was performed in 3D mode for 2 min per bed position across 6–8 bed positions.

All PET images were reconstructed using the OSEM algorithm (3 iterations, 24 subsets) with a Gaussian filter (FWHM = 4.0 mm). The reconstruction matrix was 128 × 128, and the voxel size was 4.0 × 4.0 × 4.0 mm^3^.

### Data preprocessing

A deep learning–based multimodal image registration algorithm (15) was used for image preprocessing. With CT images serving as the reference modality, both rigid and deformable registration were applied to align the ^18^F-FDG and ^18^F-octreotide PET images, ensuring precise spatial correspondence between the two tracer datasets. Following registration, all PET images were converted to standardized uptake value (SUV) maps using the formula: SUV = tissue radioactivity concentration (kBq/mL)/(injected dose (MBq)/body weight (g)). Given the marked differences in tracer biodistribution and SUV ranges between ^18^F-octreotide and ^18^F-FDG PET images, three preprocessing strategies were evaluated to minimize inter-dataset numerical discrepancies: (1) logarithmic transformation (SUV_log = log(SUV + 1)); (2) Z-score normalization; and (3) a hybrid strategy combining logarithmic transformation with subsequent Z-score normalization. The processed images were then saved in NIfTI format (.nii.gz) to facilitate downstream deep learning model training and analysis. Z-score normalization parameters (mean and standard deviation) were computed exclusively from the training cohort and then applied unchanged to the validation and test images; no information from the test cohort was used in any preprocessing step.

### Cascaded deep learning framework

A cascaded conditional generative adversarial network (cascaded conditional GAN) was developed in this study to synthesize ^18^F-FDG PET images from ^18^F-octreotide PET data. The framework was built upon the Pix2Pix architecture (16) and consisted of two sequential generator–discriminator pairs. The first stage was designed for coarse feature extraction and preliminary image synthesis, whereas the second stage focused on fine-detail refinement and enhancement.

The first-stage generator was based on a 3D U-Net architecture (17), comprising encoder and decoder pathways. The encoder included four downsampling blocks, each containing two 3 × 3 × 3 convolutional layers (stride 1, padding 1), instance normalization, and LeakyReLU activation (negative slope = 0.2), followed by a 2 × 2 × 2 max-pooling layer (stride 2). The feature channel depth increased progressively from 64 to 512. The decoder consisted of four upsampling blocks, each using a 2 × 2 × 2 transposed convolution layer (stride 2) for spatial upsampling. Skip connections were employed to fuse encoder and decoder features at corresponding resolutions, followed by two 3 × 3 × 3 convolutional layers, instance normalization, and ReLU activation. A final 1 × 1 × 1 convolution mapped the output to a single channel, and a Tanh activation function generated the normalized synthetic PET image.

The first-stage discriminator utilized a multi-scale 3D PatchGAN architecture, performing adversarial discrimination at three spatial scales (full resolution, 1/2 downsampling, and 1/4 downsampling), with identical PatchGAN structures applied at each scale. Each PatchGAN consisted of five convolutional layers with 4 × 4 × 4 kernels and strides of 2, 2, 2, 1, and 1, respectively. The corresponding channel dimensions were 64, 128, 256, 512, and 1. Instance normalization and LeakyReLU activation were applied after each layer except the final output layer. The discriminator output was a three-dimensional feature map, in which each voxel represented the authenticity probability of the corresponding image patch.

The second-stage generator was designed using a self-attention V-Net 3D architecture, which extended the first-stage U-Net by incorporating self-attention mechanisms and residual learning. The encoder pathway consisted of four residual downsampling blocks, each including two 3 × 3 × 3 convolutional layers (stride 1), instance normalization, and ReLU activation. Residual connections were applied by summing the input and output features within each block, followed by a 2 × 2 × 2 max-pooling operation. A self-attention module was introduced at the deepest encoder level (fourth layer) to model spatial attention weights and capture long-range contextual dependencies. Specifically, the self-attention mechanism reshaped feature maps into sequential representations and generated attention maps through linear transformations of Query, Key, and Value embeddings. The decoder pathway consisted of four residual upsampling blocks. Each block performed spatial upsampling using a 2 × 2 × 2 transposed convolution layer, integrated encoder features via skip connections, and subsequently applied two 3 × 3 × 3 convolutional layers with instance normalization, ReLU activation, and residual connections.

The second-stage discriminator also employed a multi-scale PatchGAN 3D architecture, with the same configuration as the first-stage discriminator.

### Model training and implementation

The cascaded deep learning model was implemented in PyTorch with the MONAI medical imaging library and trained on a single NVIDIA RTX 4090 GPU with 24 GB of memory.

The 71 paired examinations were randomly divided at the patient level into training (*n* = 43, 60.6%), validation (*n* = 14, 19.7%), and testing (*n* = 14, 19.7%) sets in a 6:2:2 ratio, with no patient contributing data to more than one subset. Both stages of the cascaded framework were trained exclusively using the training cohort, while the validation cohort was used to monitor model training and guide model selection. The held-out test cohort remained unseen during model development and was used only for final performance evaluation.

Both stages were implemented within the Pix2Pix conditional GAN framework using the least-squares GAN (LSGAN) adversarial loss, instance normalization, and Xavier weight initialization. A fixed random seed of 10 was used. The initial learning rates of the generator and discriminator were 4 × 10⁻⁴ and 2 × 10⁻⁴, respectively. The overall training objective combined the adversarial loss with a voxel-wise L1 reconstruction loss weighted by *λ*_pix2pix = 200 and bidirectional consistency terms weighted by *λ*_AB = *λ*_BA = 5.

For the second-stage parameter comparison, Group 1 was trained for 27,300 iterations with a batch size of 4, whereas Group 2 was trained for 11,000 iterations with a batch size of 1, consistent with the configurations summarized in Table 1. Model selection was performed using the validation cohort, and the test cohort was not involved in parameter selection or optimization.

**Table 1:** Experimental design for second-stage model parameter comparison.

Experimental Group	Resampling Size	Patch Size	Training Iterations	Batch Size
Group 1	128 × 128 × 256	128 × 128 × 128	27,300	4
Group 2*	256 × 256 × 512	128 × 128 × 128	11,000	1

*Optimal parameter configuration.

During inference, full-volume predictions were generated using a sliding-window strategy with a batch size of 1, an overlap ratio of 0.5, and Gaussian-weighted blending of overlapping predictions.

### Parameter comparison study

To systematically evaluate the influence of resampling resolution and patch size on image synthesis quality (Table 1), two parameter configurations were compared: a baseline configuration (Group 1; resampling size 128 × 128 × 256) and a configuration with a larger resampling size (Group 2; resampling size 256 × 256 × 512); the patch size was fixed at 128 × 128 × 128 for both. Both models were trained exclusively on the training cohort (*n* = 43), and the validation cohort (*n* = 14) was used to monitor training and guide model selection; the held-out test cohort (*n* = 14) remained completely unseen during training and parameter optimization and was used only for the final evaluation, in which both configurations were assessed on the identical 14 test patients. Because the two configurations also differed in training iterations and batch size (Table 1), the comparison reflects the overall parameter configuration rather than the isolated effect of resampling resolution. In this framework, the resampling size determined the spatial resolution of the network input, whereas the patch size defined the local receptive region processed by the model.

### Image quality assessment

The quality of synthesized images was quantitatively evaluated using five objective metrics (18, 19). The Structural Similarity Index Measure (SSIM) assesses similarity between synthesized and reference images from the perspectives of luminance, contrast, and structural consistency, with values ranging from 0 to 1 and values closer to 1 indicating greater similarity. The Peak Signal-to-Noise Ratio (PSNR) quantifies pixel-wise differences between synthesized and reference images and is expressed in decibels (dB), with higher values indicating superior image quality. The Mean Squared Error (MSE) represents the average squared difference between corresponding pixels in synthesized and reference images, with lower values indicating reduced reconstruction error. The Mean Absolute Error (MAE) represents the average absolute difference between corresponding pixels in synthesized and reference images, with lower values indicating better synthesis accuracy. Normalized Mutual Information (NMI) is an information-theoretic metric used to assess the statistical dependence and shared information between two images, with higher values indicating stronger information consistency.

For qualitative evaluation, three nuclear medicine physicians with more than 5 years of diagnostic experience independently performed visual assessments of the synthesized images using a 5-point Likert scale: 1 = poor (poor image quality, not suitable for diagnosis); 2 = fair (suboptimal image quality with blurred lesion visualization); 3 = moderate (acceptable image quality with adequate lesion visualization); 4 = good (good image quality with clear lesion visualization); and 5 = excellent (excellent image quality, nearly indistinguishable from real images). The physicians were blinded to whether the images were synthesized and independently assigned scores, and the average score was used as the final evaluation result. Images were presented in randomized order; readers were blinded to image type, patient identity, and clinical information, and scored each image independently without access to the paired real reference image. Only synthetic images were scored. Inter-reader agreement was calculated from the raw individual scores using a two-way random-effects, absolute-agreement, average-measures intraclass correlation coefficient (ICC(2,3)). The Likert score reflects perceived image quality and visual similarity and was not intended to measure diagnostic performance.

Semi-quantitative evaluation was performed to assess the consistency of metabolic quantification between synthesized and real images. The mean standardized uptake value (SUVmean) was measured using a standard volume of interest (VOI) positioned in the mid-right hepatic lobe (3-cm spherical VOI excluding major blood vessels). For lesion-level quantitative analysis, tumor lesions were semi-automatically delineated on PET images using a 41% SUVmax threshold with reference to the co-registered CT images. In the 14 patients of the test set, lesion size (long and short diameters), SUVmax, SUVmean, and SUVpeak were measured on both real ^18^F-FDG and synthetic ^18^F-FDG images; for patients with multiple lesions, the largest lesion was selected for analysis. SUVpeak could not be calculated in one patient because the delineated lesion volume was smaller than the minimum volume required for SUVpeak computation; therefore, SUVpeak analysis was performed in 13 patients. The identical semi-automatic delineation procedure (41% SUVmax threshold with reference to the co-registered CT) was applied independently to the real and synthetic ^18^F-FDG images; no lesion VOI was transferred between image types.

### Statistical analysis

All statistical analyses were performed using Python 3.9 and R 4.2. Continuous variables were expressed as mean ± standard deviation (Mean ± SD). Baseline characteristics among the training, validation, and testing cohorts were compared. Continuous variables (age, BMI, height, and weight) were evaluated for normality using the Shapiro–Wilk test and for homogeneity of variance using Levene's test. Variables satisfying parametric assumptions were compared across the three cohorts using one-way analysis of variance (ANOVA), with F statistics and corresponding *P* values reported. Variables that did not meet parametric assumptions were analyzed using the Kruskal–Wallis H test, with H statistics reported. Categorical variables (sex and NET subtypes) were expressed as frequencies and percentages and analyzed using the chi-square (*χ*^2^) test or Fisher's exact test, with *χ*^2^ values and *P* values reported. Comparisons among the three preprocessing strategies were performed using repeated-measures ANOVA, with the Friedman test used as a non-parametric sensitivity analysis. *Post-hoc* paired comparisons were performed with Holm correction for multiple comparisons. Comparisons between the two parameter configurations were conducted using paired-samples t-tests because both configurations were evaluated on the identical 14 test patients. Statistical significance was defined as *P* < 0.05. Effect sizes were quantified using Cohen's d. Ninety-five percent confidence intervals were calculated for the primary between-configuration mean differences. For lesion-level and liver quantitative agreement analyses between synthetic and real ^18^F-FDG images, Pearson correlation coefficients and Spearman rank correlation coefficients were calculated for lesion SUVmax, SUVmean, SUVpeak, and liver SUVmean. Ninety-five percent confidence intervals (CIs) for Pearson correlation coefficients were calculated using Fisher's z transformation. Lin's concordance correlation coefficient (CCC) was additionally calculated to assess agreement between synthetic and real measurements. Paired-samples t-tests were used to compare corresponding measurements. Bland–Altman analysis was performed to quantify the mean bias and 95% limits of agreement (LoA), and 95% CIs were calculated for both the mean bias and the lower and upper LoA.

## Results

### Patient demographic characteristics

A total of 71 tumor patients were included in this study, comprising 43 patients (60.6%) in the training set, 14 patients (19.7%) in the validation set, and 14 patients (19.7%) in the test set. No statistically significant differences were observed among the three groups in terms of age, sex, BMI, or tumor type distribution (*P* > 0.05), indicating good comparability. Detailed demographic characteristics of the patients are presented in Table 2.

**Table 2:** Patient demographic characteristics.

Characteristic	Training Set (*n* = 43)	Validation Set (*n* = 14)	Test Set (*n* = 14)	Statistic	*P* value
Age	56.3 ± 14.5	56.0 ± 13.8	58.4 ± 12.8	F = 0.137	0.872
Sex (male/female)	25/18	9/5	8/6	*χ*^2^ = 0.194	0.907
BMI (kg/m^2^)	22.5 ± 2.9	22.3 ± 2.5	23.5 ± 2.8	F = 0.811	0.449
Height (m)	1.67 ± 0.07	1.68 ± 0.06	1.68 ± 0.06	F = 0.193	0.825
Weight (kg)	63.2 ± 10.6	63.8 ± 9.5	64.3 ± 11.2	F = 0.063	0.939
Pancreatic NETs	15 (34.9%)	5 (35.7%)	4 (28.6%)	χ^2^ = 0.217	0.897
Rectal NETs	4 (9.3%)	1 (7.1%)	1 (7.1%)	χ^2^ = 0.102	0.950
Gastric NETs	2 (4.7%)	1 (7.1%)	1 (7.1%)	χ^2^ = 0.198	0.906
Liver NETs	2 (4.7%)	1 (7.1%)	1 (7.1%)	χ^2^ = 0.198	0.906
Other NETs	7 (16.3%)	2 (14.3%)	3 (21.4%)	χ^2^ = 0.284	0.867
Non-NET tumors	13 (30.2%)	4 (28.6%)	4 (28.6%)	χ^2^ = 0.022	0.989

BMI, body mass index; NETs, neuroendocrine tumors. No statistically significant differences were observed among the three groups in age, sex, BMI, or tumor type distribution (*P* > 0.05).

### Impact of data preprocessing strategies on synthesis quality

The quantitative evaluation results of the first-stage model using three different data preprocessing strategies in the held-out test cohort (*n* = 14) are presented in Table 3. Representative images illustrating the synthesis process from ^18^F-OCT PET to ^18^F-FDG PET are shown in Figure 2. The combined strategy (logarithmic transformation + normalization) consistently achieved the best performance across all five evaluation metrics. The combined method achieved an SSIM of 0.8454 ± 0.0328, compared with 0.7831 ± 0.0337 for the normalization method and 0.7438 ± 0.0362 for the logarithmic transformation method. The corresponding PSNR values were 27.33 ± 3.36, 25.47 ± 3.27, and 24.17 ± 2.96 dB, respectively. Repeated-measures ANOVA demonstrated significant differences among the three preprocessing strategies for all five metrics (all *P* < 0.001). Friedman sensitivity analyses yielded consistent results (all *P* < 0.001), and *post-hoc* paired comparisons with Holm correction showed significant differences between all three preprocessing strategies for all five metrics (all adjusted *P* < 0.001). The comparison of synthesis performance among the three preprocessing strategies is illustrated in Figure 3.

**Table 3:** Comparison of synthesis performance among three data preprocessing methods (test set, *n* = 14).

Metric	Combined	Normalization	Log	Repeated-measures ANOVA	Friedman
SSIM	0.8454 ± 0.0328	0.7831 ± 0.0337	0.7438 ± 0.0362	F(2,26) = 560.50, *P* < 0.001	χ^2^ = 28.00, *P* < 0.001
PSNR (dB)	27.33 ± 3.36	25.47 ± 3.27	24.17 ± 2.96	F(2,26) = 330.08, *P* < 0.001	χ^2^ = 28.00, *P* < 0.001
MSE	0.4986 ± 0.1179	0.5323 ± 0.1235	0.5568 ± 0.1267	F(2,26) = 212.02, *P* < 0.001	χ^2^ = 28.00, *P* < 0.001
MAE	0.2119 ± 0.0117	0.2281 ± 0.0115	0.2400 ± 0.0141	F(2,26) = 174.69, *P* < 0.001	χ^2^ = 28.00, *P* < 0.001
NMI	1.2060 ± 0.0461	1.1230 ± 0.0425	1.0712 ± 0.0402	F(2,26) = 351.80, *P* < 0.001	χ^2^ = 28.00, *P* < 0.001

Comparisons among the three preprocessing strategies were performed using repeated-measures ANOVA, with the Friedman test as a non-parametric sensitivity analysis. *Post-hoc* paired comparisons with Holm correction showed significant differences between all three preprocessing strategies for all five metrics (all adjusted *P* < 0.001).

**Figure 2:**
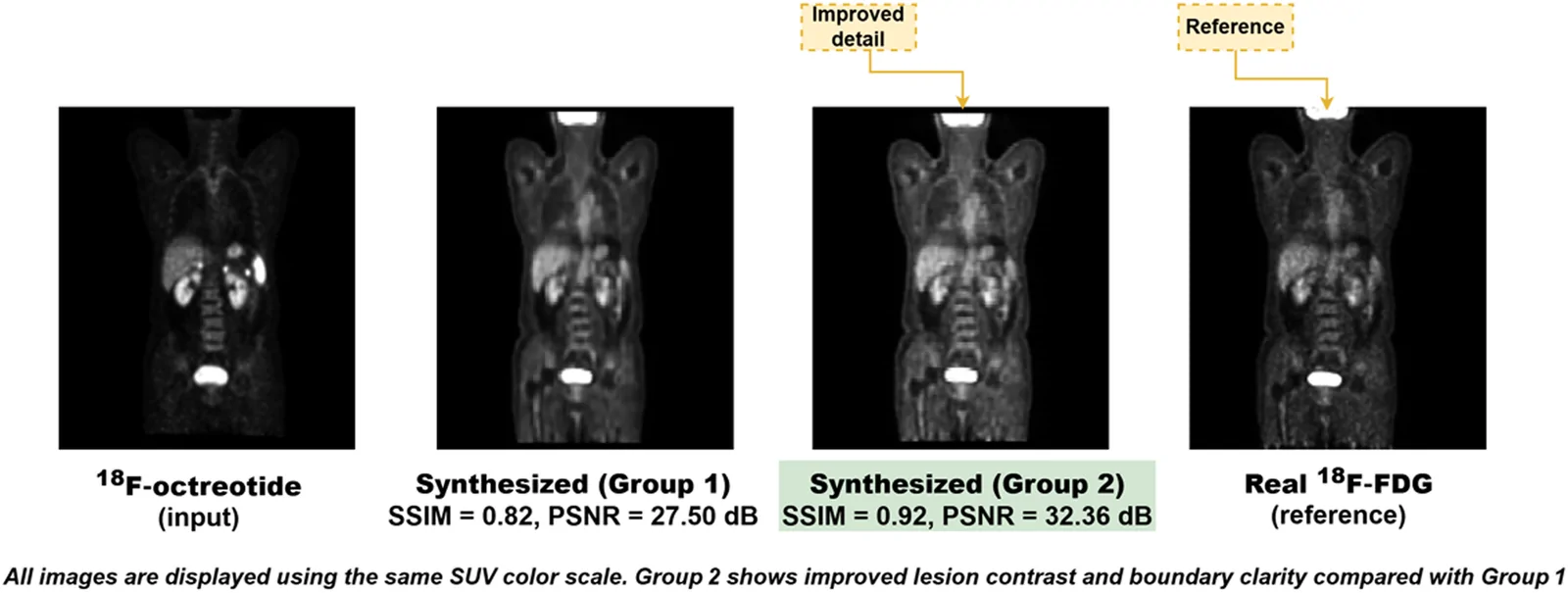
Comparison of synthesis performance between group 1 and group 2 configurations. Representative synthesis results from the test set. The four columns show (from left to right): the original ^18^F-octreotide PET input, synthetic ^18^F-FDG PET images generated by Group 1 (resampling 128 × 128 × 256) and Group 2 (resampling 256 × 256 × 512), and the real ^18^F-FDG PET reference. Below each synthetic image, the corresponding SSIM and PSNR values are reported (cohort means — Group 1: SSIM 0.74, PSNR 15.34 dB; Group 2: SSIM 0.89, PSNR 28.15 dB). Compared with Group 1, Group 2 produced images with sharper lesion boundaries, better preserved metabolic details, and higher visual similarity to the real ^18^F-FDG images. All images are displayed using the same SUV color scale.

**Figure 3:**
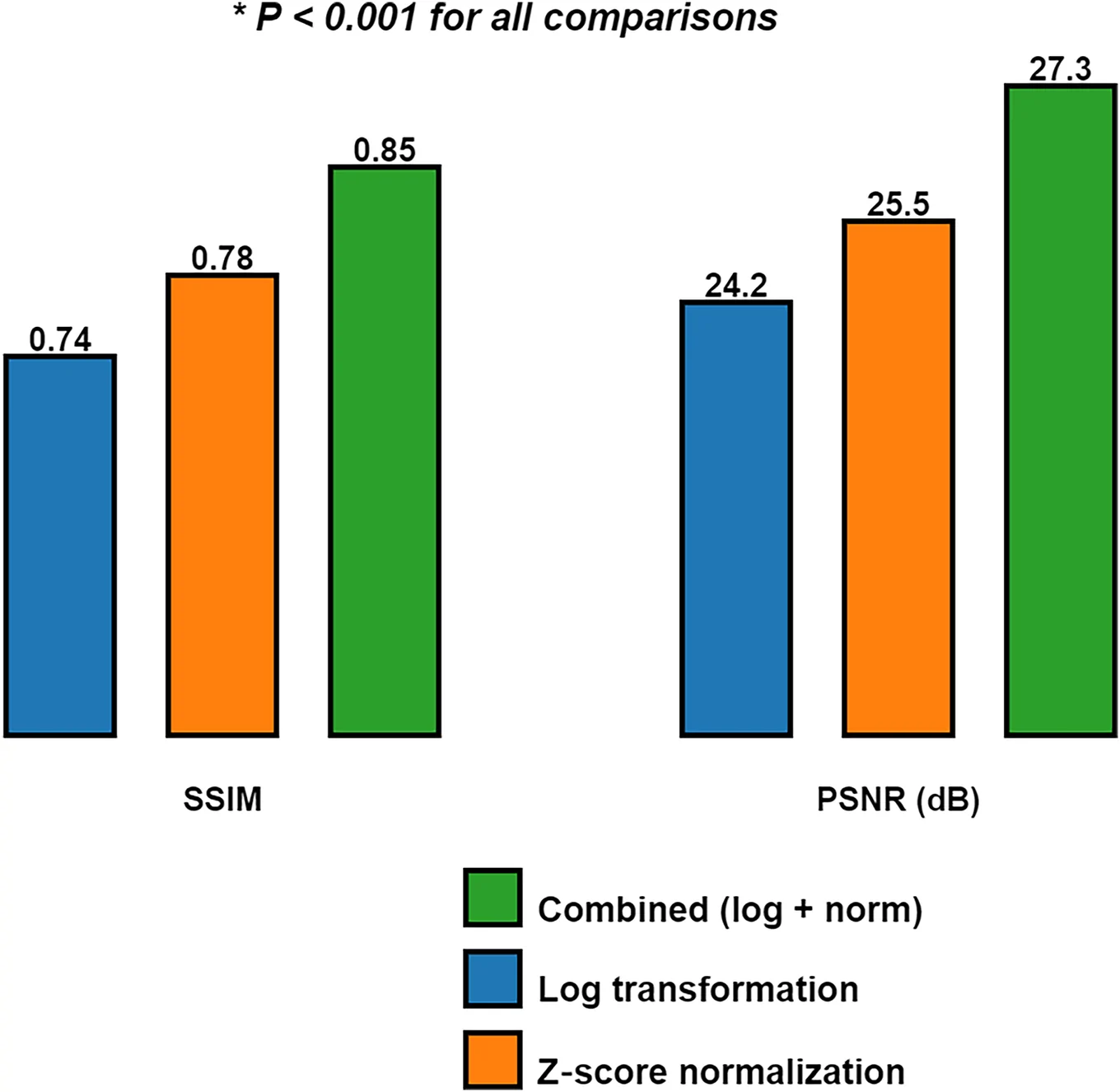
Performance of the three preprocessing strategies in the held-out test set (*n* = 14). The bar chart shows the mean SSIM (left) and PSNR (right) for logarithmic transformation, Z-score normalization, and the combined strategy (log transformation + normalization). The combined strategy achieved the highest SSIM (0.8454) and PSNR (27.33 dB). Repeated-measures ANOVA showed significant differences among the three strategies, and Holm-corrected *post-hoc* paired comparisons confirmed significant pairwise differences (all adjusted *P* < 0.001).

### Optimization of second-stage parameters for synthesis performance

Based on the combined preprocessing strategy, the synthesis performance of the two parameter configurations was compared on the identical held-out test cohort (*n* = 14) using paired-samples t-tests (Table 4). Group 2 (resampling size: 256 × 256 × 512; patch size: 128 × 128 × 128) significantly outperformed Group 1 (resampling size: 128 × 128 × 256; patch size: 128 × 128 × 128) in SSIM, PSNR, MSE, and MAE. Group 2 achieved an SSIM of 0.8854 ± 0.0455, representing an absolute increase of 0.1423 (95% CI: 0.1089–0.1757) and a relative improvement of 19.1% compared with Group 1 (0.7431 ± 0.0239) (t = −9.21, *P* < 0.001, Cohen's d = 2.46). The PSNR reached 28.15 ± 3.35 dB, an absolute increase of 12.81 dB (95% CI: 11.03–14.59 dB) and a relative improvement of 83.5% over Group 1 (15.34 ± 0.66 dB) (t = −15.54, *P* < 0.001, Cohen's d = 4.15). MSE decreased by 80.8% (t = 20.89, *P* < 0.001, Cohen's d = 5.58), and MAE decreased by 63.3% (t = 15.38, *P* < 0.001, Cohen's d = 4.11). NMI did not differ significantly between the two configurations (1.2943 ± 0.0535 vs. 1.2738 ± 0.0212; t = −1.14, *P* = 0.276, Cohen's d = 0.30). The relative improvements of Group 2 over Group 1 across the five objective metrics are presented in Figure 4.

**Table 4:** Comparative synthesis performance of two parameter configurations (test set, *n* = 14).

Metric	Group 1 (*n* = 14)	Group 2 (*n* = 14)	t value	*P* value	Cohen's d
SSIM	0.7431 ± 0.0239	0.8854 ± 0.0455	−9.21	<0.001*	2.46
PSNR (dB)	15.34 ± 0.66	28.15 ± 3.35	−15.54	<0.001*	4.15
MSE	0.0102 ± 0.0015	0.0020 ± 0.0013	20.89	<0.001*	5.58
MAE	0.0389 ± 0.0047	0.0143 ± 0.0055	15.38	<0.001*	4.11
NMI	1.2738 ± 0.0212	1.2943 ± 0.0535	−1.14	0.276	0.30

SSIM, structural similarity index measure; PSNR, peak signal-to-noise ratio; MSE, mean squared error; MAE, mean absolute error; NMI = normalized mutual information. Group 2 versus Group 1 comparisons were performed using paired-samples t-tests because both configurations were evaluated on the identical 14 test patients; Cohen's d was calculated for paired designs.

*P < 0.05.

**Figure 4:**
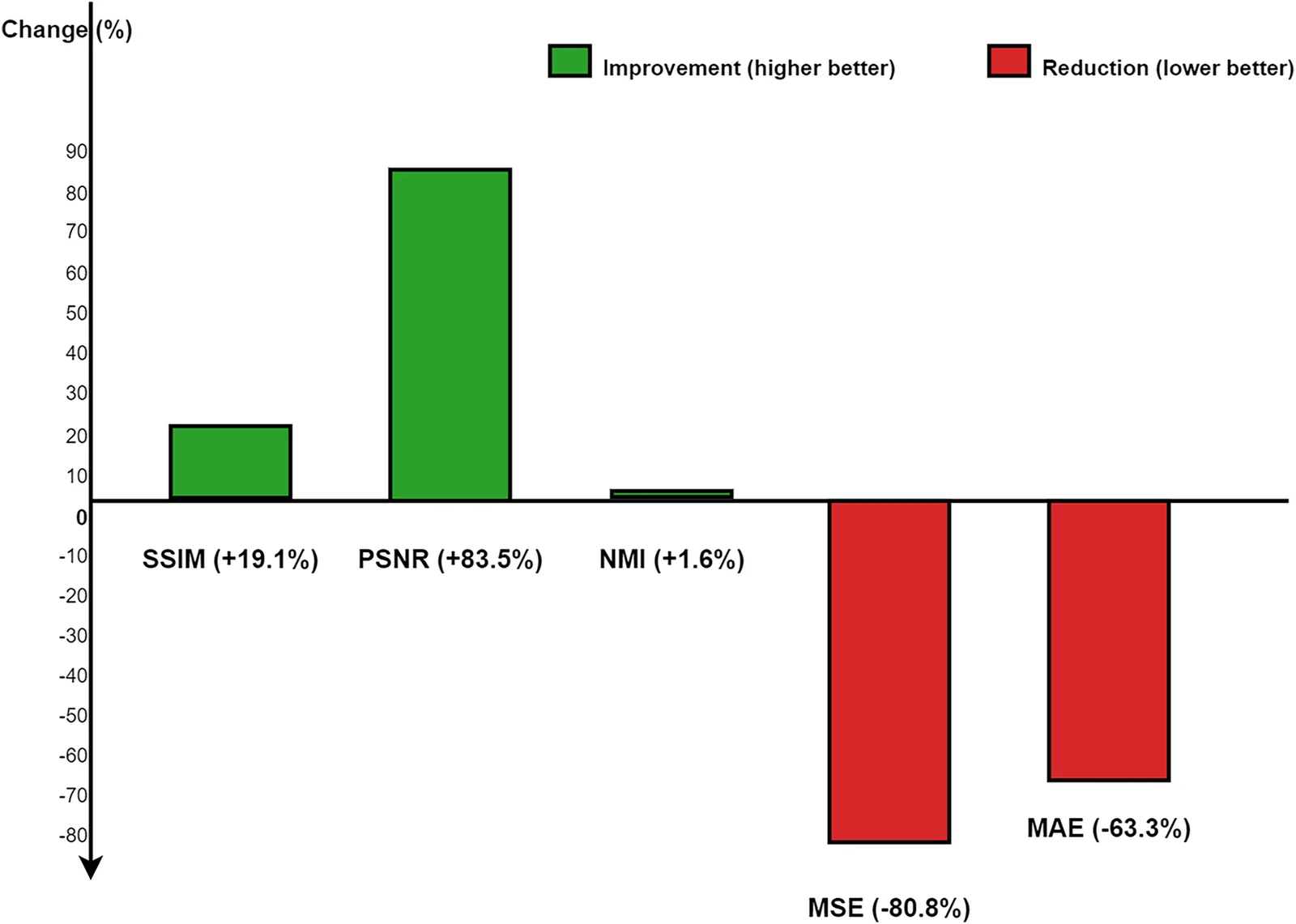
Relative improvement of group 2 over group 1 across five objective metrics. Positive percentages (SSIM, PSNR, NMI) indicate higher values are better; negative metrics (MSE, MAE) are presented as percentage reduction. Group 2 achieved relative improvements of +19.1% for SSIM and +83.5% for PSNR, while MSE and MAE decreased by 80.8% and 63.3%, respectively (all *P* < 0.001, paired-samples t-test); the NMI difference was not significant (+1.6%, *P* = 0.276). Effect sizes (Cohen's d) ranged from 0.30 to 5.58.

### Qualitative evaluation results

Three nuclear medicine physicians performed blinded visual assessments of the 14 synthesized test images generated by the Group 2 model. The mean Likert 5-point score was 4.53 ± 0.19 (range: 4.2–4.8), indicating excellent image quality. All 14 images received scores above 4 points (good or excellent), and 10 cases (71.4%) were rated as 5 points (excellent). Interobserver agreement among the three physicians was good (ICC = 0.89, 95% CI: 0.82–0.94).

### Lesion-level and liver quantitative consistency analysis of SUV

Lesion-level and liver quantitative agreement between synthetic and real ^18^F-FDG images in the test set is summarized in Table 5, and the corresponding scatter plots and Bland–Altman plots are shown in Figure 5. At the lesion level (*n* = 14), correlations between synthetic and real ^18^F-FDG were almost perfect for all three metrics: SUVmax (Pearson r = 1.0000, *P* < 0.001; Spearman *ρ* = 1.0000), SUVmean (r = 0.9995, *P* < 0.001; *ρ* = 0.9945), and SUVpeak (*n* = 13; r = 0.9991, *P* < 0.001; *ρ* = 0.9986) (the top row of Figure 5). Paired t-tests revealed no significant differences between synthetic and real images for SUVmax (*P* = 0.816), SUVmean (*P* = 0.762), or SUVpeak (*P* = 0.205). Bland–Altman analysis demonstrated minimal bias for SUVmax (0.004; 95% LoA: −0.118 to 0.126), SUVmean (0.005; 95% LoA: −0.107 to 0.117), and SUVpeak (0.073; 95% LoA: −0.311 to 0.457), with all data points falling within the 95% LoA (the bottom row of Figure 5). For liver background quantification, synthetic and real images showed SUVmean values of 3.39 ± 0.75 and 3.40 ± 0.75, respectively (r = 0.9933, *P* < 0.001; *ρ* = 0.9780), with no significant difference on the paired t-test (bias = −0.001; *P* = 0.967; 95% LoA: −0.172 to 0.170). A representative lesion-level comparison is illustrated in Figure 6. These findings indicate high quantitative agreement between synthetic and real ^18^F-FDG PET at the lesion and reference-organ level in this exploratory, single-center pilot cohort; the clinical acceptability and precision of the observed agreement estimates should be confirmed in larger cohorts. Exploratory SSTR/FDG quadrant analysis. Using the lesion-to-liver SUVmax ratio (threshold > 1.0) on the real ^18^F-octreotide and ^18^F-FDG images, test-cohort target lesions were classified as SSTR-positive/FDG-positive (*n* = 6), SSTR-positive/FDG-negative (*n* = 5), and SSTR-negative/FDG-negative (*n* = 3); no SSTR-negative/FDG-positive lesion was present in this cohort. The number of lesions in the biologically discordant quadrants was therefore too small for a statistically meaningful subgroup analysis of synthesis performance. Lesion long diameters on real ^18^F-FDG images ranged from 2.3 to 102.5 mm (median: 22.8 mm); five lesions (35.7%) were smaller than 20 mm.

**Table 5:** Lesion-level and liver quantitative agreement between real and synthetic ^18^F-FDG PET images in the test set (Pearson correlation, paired t-test, and bland–altman analysis).

Statistic	Lesion SUVmax	Lesion SUVmean	Lesion SUVpeak	Liver SUVmean
n	14	14	13	14
Real FDG (mean ± SD)	4.90 ± 5.06	2.61 ± 1.75	4.41 ± 4.50	3.40 ± 0.75
Synthetic FDG (mean ± SD)	4.90 ± 5.02	2.61 ± 1.74	4.48 ± 4.49	3.39 ± 0.75
Pearson r (95% CI)	1.0000 (0.9999–1.0000)	0.9995 (0.9984–0.9998)	0.9991 (0.9967–0.9997)	0.9933 (0.9782–0.9979)
*P* value (Pearson correlation)	<0.001	<0.001	<0.001	<0.001
Spearman *ρ*	1.0000	0.9945	0.9986	0.9780
Lin's CCC	0.9999	0.9995	0.9989	0.9933
*P* value (paired t-test)	0.816	0.762	0.205	0.967
Bias (synthetic−real) (95% CI)	0.004 (−0.032–0.040)	0.005 (−0.028–0.038)	0.073 (−0.046–0.191)	−0.001 (−0.051–0.049)
SD of differences	0.062	0.057	0.196	0.087
95% LoA lower limit (95% CI)	−0.118 (−0.182 - −0.054)	−0.107 (−0.166 - −0.049)	−0.311 (−0.522 - −0.101)	−0.172 (−0.261 - −0.082)
95% LoA upper limit (95% CI)	0.126 (0.062–0.190)	0.117 (0.058–0.175)	0.457 (0.246–0.667)	0.170 (0.080–0.259)

Pearson r ≥ 0.993 (*P* < 0.001) and Spearman ρ ≥ 0.978 were observed for all four parameters; paired t-tests showed no significant differences between synthetic and real images (all *P* > 0.05). The absolute Bland–Altman bias was ≤ 0.073 SUV, and all difference points lay within the 95% limits of agreement. SUVpeak was calculable in 13 of the 14 test patients. Liver background quantification (SUVmean) was highly consistent between real and synthetic images, further confirming reference-organ fidelity. SD,  standard deviation; LoA, limits of agreement.

**Figure 5:**
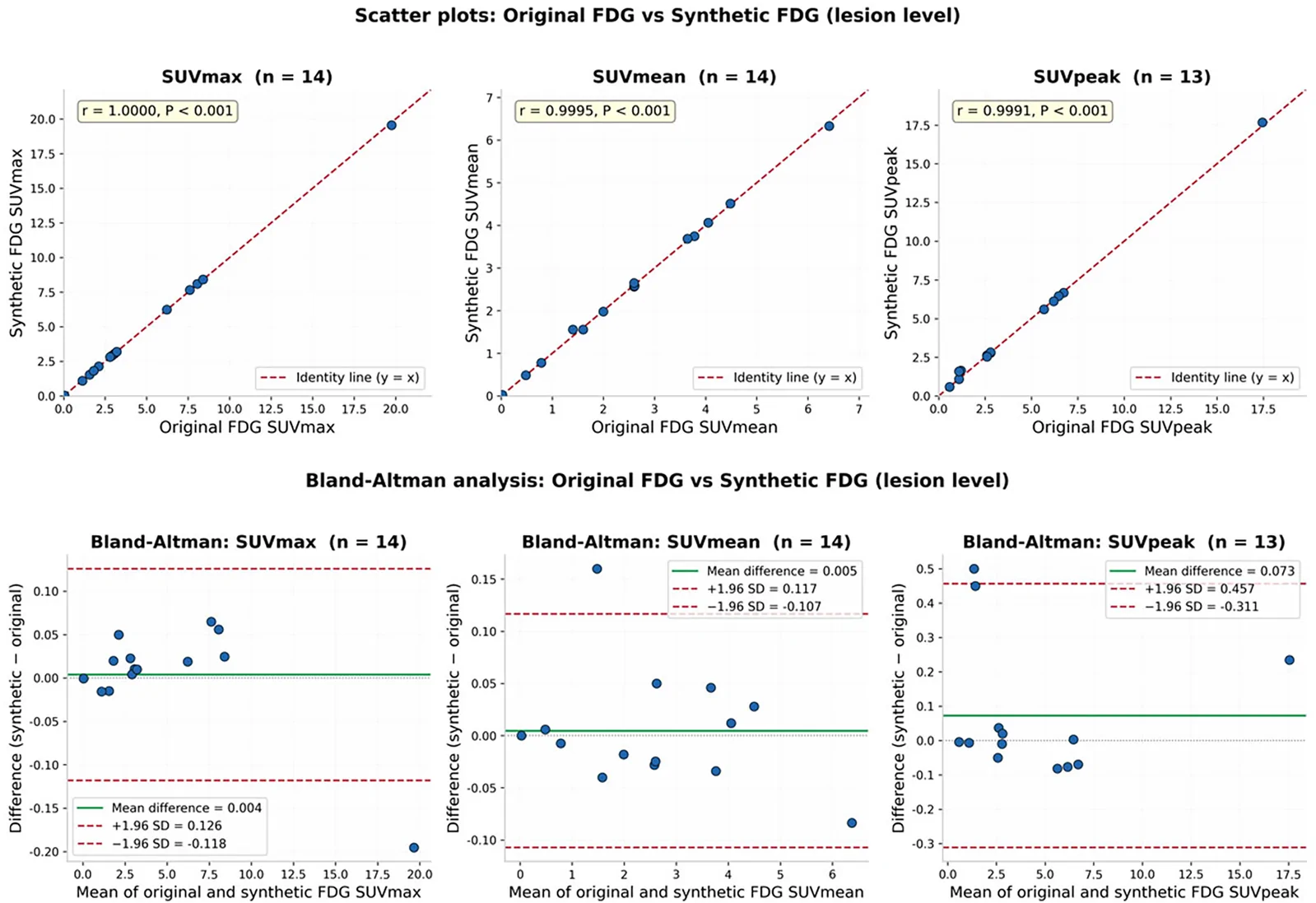
Lesion-level quantitative agreement between synthetic and real ^18^F-FDG PET images in the test set. Top row: scatter plots of synthetic versus real measurements for lesion SUVmax and SUVmean (n = 14) and SUVpeak (n = 13); the dashed line indicates the identity line (y = x), and Pearson correlation coefficients are annotated for each parameter. Bottom row: Bland-Altman plots for the same lesion parameters; the solid line indicates the mean bias, and the dashed lines indicate the 95% limits of agreement. All three lesion measurements showed almost perfect correlation with minimal bias, and all data points fell within the 95% limits of agreement.

**Figure 6:**
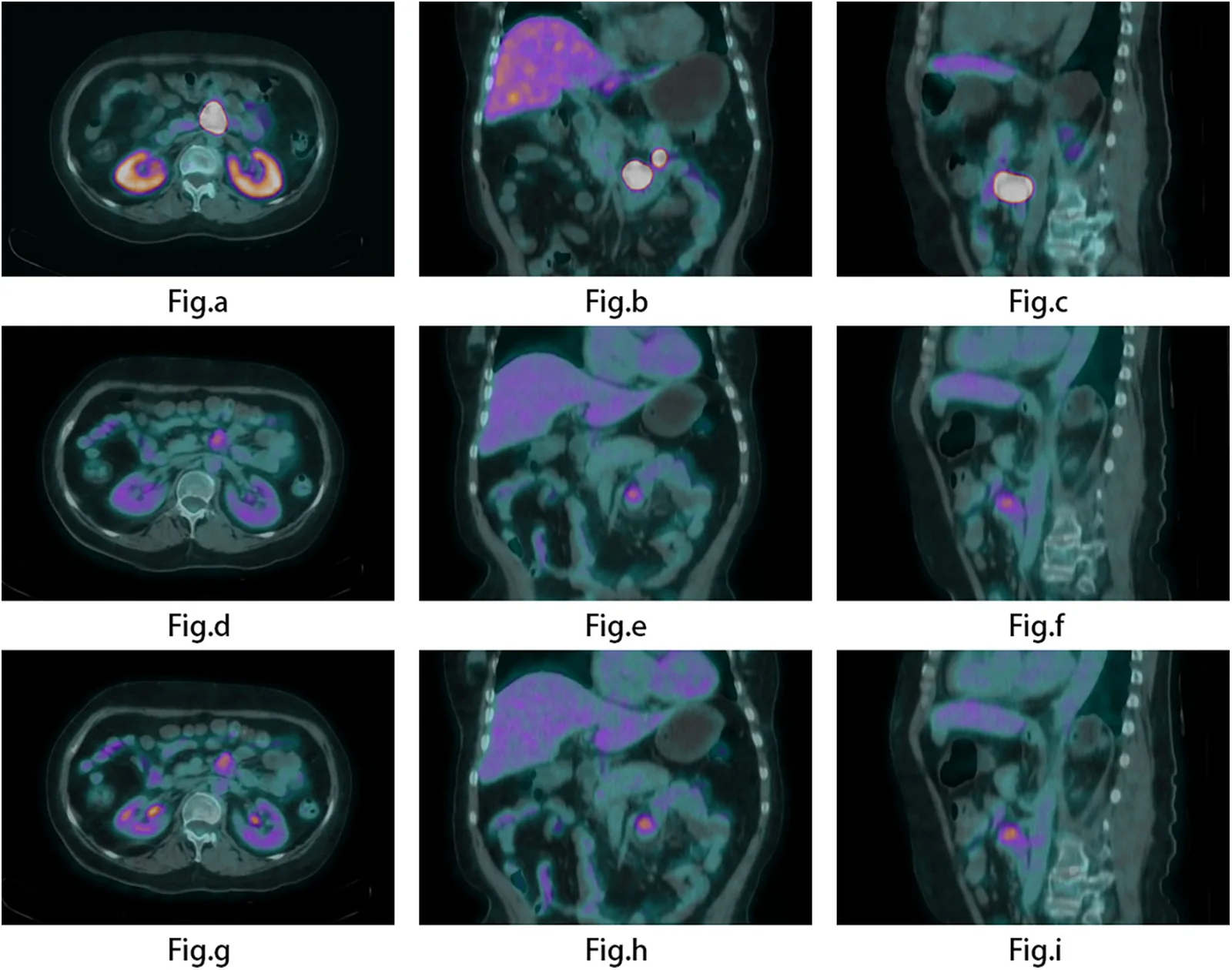
Lesion-level visual comparison among ^18^F-octreotide PET, synthetic ^18^F-FDG PET, and real ^18^F-FDG PET images. Representative axial, coronal, and sagittal views are shown. **(A–C)** ^18^F-octreotide PET images; **(D–F)** synthetic ^18^F-FDG PET images generated from ^18^F-octreotide PET; **(G–I)** real ^18^F-FDG PET images. The highlighted region indicates the representative lesion.

## Discussion

This study developed and evaluated a cascaded deep learning–based framework for cross-tracer synthesis of ^18^F-FDG PET images from ^18^F-octreotide PET images. After systematic optimization of preprocessing and network input parameters, the synthesized images achieved high structural fidelity, quantitative agreement, and visual quality in the test cohort. The principal finding is that task decoupling across two supervised stages can support cross-biological tracer translation with high quantitative fidelity in this exploratory, single-center pilot cohort.

The cascaded architecture addresses the complexity of cross-tracer mapping through a task-decoupling strategy. The first-stage 3D U-Net performs anatomically constrained coarse synthesis, establishing organ-level localization and background uptake, whereas the second-stage self-attention V-Net 3D refines FDG-specific metabolic features from the preliminary output. The self-attention module captures long-range spatial dependencies, which may improve anatomical consistency and lesion boundary delineation. By decomposing cross-domain mapping and detail reconstruction into two sequential optimization steps, the cascaded framework reduces the learning burden at each stage and provides a practical strategy for overcoming the performance limitations of single-stage networks (14).

Preprocessing and input parameterization were important determinants of synthesis quality. SUV values in PET images may span a wide dynamic range, and focal regions with very high uptake can disproportionately influence gradient updates. Logarithmic transformation compressed this dynamic range, whereas Z-score normalization standardized the intensity distribution and promoted stable network convergence. To our knowledge, this study is among the first to systematically evaluate their combined use in cross-tracer PET synthesis. The combined strategy yielded significantly higher SSIM and PSNR values than either preprocessing method alone (*P* < 0.001), supporting its use as a robust preprocessing pipeline for this task. The configuration with a larger resampling size (256 × 256 × 512) achieved the better overall synthesis performance. Because the two configurations differed not only in resampling size but also in training iterations and batch size, the observed improvement should be attributed to the overall parameter configuration rather than to resampling resolution alone; a controlled ablation varying one parameter at a time is planned as future work. These findings provide practical guidance for configuring cascaded PET synthesis models.

Lesion-level agreement analysis provides stronger evidence of clinical relevance than global image similarity or liver background quantification alone. Hepatic uptake is physiologically homogeneous and shows limited inter-individual variability; consequently, liver SUVmean agreement may remain high even when focal lesions are imperfectly synthesized. In contrast, lesion-level metrics directly evaluate the regions most relevant to diagnosis, staging, and treatment-response assessment. In this study, synthetic and real ^18^F-FDG images showed almost perfect correlations for lesion SUVmax, SUVmean, and SUVpeak (Pearson r ≥ 0.999), with minimal Bland–Altman bias (≤ 0.073 SUV) and all observations within the 95% limits of agreement. These results indicate that the cascaded framework preserves both reference-organ uptake and focal lesion quantification in the test cohort.

The potential clinical value of this approach lies in reducing the burden of dual-tracer imaging while retaining complementary molecular information. The optimized configuration achieved an SSIM of 0.8854 and a PSNR of 28.15 dB, and physician ratings averaged 4.53 on a 5-point scale in the test cohort. Synthetic images also showed close agreement with real images for liver background and lesion-level SUV measurements. Conventional dual-tracer assessment requires two PET/CT examinations on separate days, increasing cost, workflow complexity, and cumulative radiation exposure. A previous whole-body ^18^F-FDG PET/CT dosimetry study reported an effective dose of approximately 14 mSv per examination (7); therefore, omitting a second acquisition may be particularly relevant for patients requiring longitudinal surveillance, such as those with multiple endocrine neoplasia type 1. In addition, preserving the acquired SSTR image while generating an FDG-like metabolic map may facilitate direct spatial comparison of SSTR expression and glucose metabolism. This dual-parameter assessment could support molecular phenotyping of NETs and assist in evaluating candidates for peptide receptor radionuclide therapy (4, 5, 20). It should be emphasized that the present study demonstrates image-synthesis performance rather than clinical utility: no diagnostic-accuracy, staging, treatment-decision, or outcome analyses were performed, and the Likert ratings reflect image quality and visual similarity rather than diagnostic equivalence. Prospective studies are nevertheless required to determine whether synthesized images affect diagnostic confidence, treatment selection, or patient outcomes.

This study has several limitations. First, the sample size was limited (71 cases), and the study was conducted retrospectively at a single center; accordingly, this study should be regarded as an exploratory pilot investigation, and the precision of the agreement estimates is limited by the small test cohort. Although strict eligibility criteria and patient-level data splitting were applied, generalizability to larger multicenter cohorts remains uncertain. PET synthesis performance may be sensitive to scanner manufacturer and model, acquisition time, injected activity, uptake time, reconstruction algorithm and parameters, spatial resolution, patient body habitus, and disease distribution (21, 22); although two Philips PET/CT systems were used, the test cohort was too small for a meaningful scanner-specific subgroup analysis. Second, synthesis accuracy and boundary delineation for small lesions require further improvement because these lesions are susceptible to low signal-to-noise ratios and partial-volume effects. In addition, only the largest lesion per patient was analyzed, which may introduce selection bias and overestimate agreement, because smaller lesions and lesions with heterogeneous uptake are likely to be the most challenging synthesis targets. Third, although lesion-level analyses were included, quantitative evaluation of normal background uptake was primarily limited to the liver; systematic assessment of other organs and of different lesion types is still needed. Fourth, the biological differences between ^18^F-octreotide and ^18^F-FDG—SSTR expression versus glucose metabolism—create intrinsic differences in lesion contrast and physiological distribution. Synthesis performance may therefore be limited in dedifferentiated lesions with low SSTR expression but high FDG uptake. Fifth, this problem-driven study did not systematically benchmark the proposed framework against the full range of contemporary generative architectures; broader comparisons should be conducted in larger, harmonized datasets. Sixth, stratified analysis according to the four SSTR/FDG concordance categories was not feasible because the number of lesions in each discordant quadrant was too small for statistically meaningful subgroup evaluation; in particular, no SSTR-negative/FDG-positive lesion was present in the test cohort, and the present results may therefore primarily reflect patients in whom the biological relationship between the two tracers is learnable. Seventh, the framework uses PET images as the sole input and does not exploit anatomical information from the accompanying CT; incorporating CT as an additional input channel may further improve lesion localization and boundary delineation. The PET-only design was chosen deliberately so that synthesis depends solely on the molecular input, with CT used only as the spatial reference for inter-tracer registration; a systematic PET-only versus PET + CT comparison is warranted in future work. Finally, global image-quality metrics such as SSIM, PSNR, MSE, MAE, and NMI, although standard and complementary, may mask clinically important errors in small or high-uptake lesions and should therefore be complemented by lesion-targeted analyses in future studies, including contrast recovery, lesion-size-stratified SUV bias, and metabolic tumour volume agreement.

Future studies should focus on the following directions: (1) conducting multicenter, prospective studies with larger sample sizes to validate generalizability and clinical diagnostic performance; (2) benchmarking the proposed framework against contemporary generative architectures using harmonized datasets and evaluation protocols; (3) developing small-lesion enhancement strategies to improve detection and visualization of micro-lesions; (4) establishing whole-organ SUV calibration models across multiple organs and disease types; and (5) evaluating prospectively whether synthesized images influence NET staging, treatment selection, and clinical decision-making pathways.

## Conclusion

In this study, a cascaded deep learning framework was established for synthesizing ^18^F-FDG PET images from ^18^F-octreotide PET data, and its preprocessing and input-parameter configurations were systematically optimized. The combined logarithmic transformation and normalization strategy outperformed individual preprocessing methods, and the optimized configuration achieved significantly better structural similarity and pixel-level accuracy than the baseline configuration in the held-out test cohort. Lesion-level analysis demonstrated almost perfect agreement between synthetic and real images for SUVmax, SUVmean, and SUVpeak, supporting the image-synthesis feasibility of this approach as an auxiliary imaging strategy. Multicenter prospective validation and broader architectural benchmarking are required before routine clinical implementation.

## Data Availability

The raw data supporting the conclusions of this article will be made available by the authors, without undue reservation.
