# Supplementary material for: Cascaded deep learning for synthesis of 18F-FDG PET from 18F-octreotide: model parameter optimization and performance evaluation
Source: Front Nucl Med. 2026 Oct 16;6:1920721. doi: 10.3389/fnume.2026.1920721 (PMC13719449; doi:10.3389/fnume.2026.1920721)
Supplement: Supplementary file 1 [file Supplementaryfile1.docx]

**Supplementary Table S1. Lesion-level quantitative PET measurements in the test cohort**

| **Patient ID** | **OCT SUVmax** | **OCT SUVmean** | **OCT SUVpeak** | **Real FDG SUVmax** | **Real FDG SUVmean** | **Real FDG SUVpeak** | **Synthetic FDG SUVmax** | **Synthetic FDG SUVmean** | **Synthetic FDG SUVpeak** | **Real FDG liver SUVmean** | **Synthetic FDG liver SUVmean** |
| --- | --- | --- | --- | --- | --- | --- | --- | --- | --- | --- | --- |
| P1 | 3.607 | 2.478 | 3.252 | 8.042 | 4.051 | 6.736 | 8.098 | 4.063 | 6.667 | 2.405 | 2.400 |
| P2 | 61.226 | 31.894 | 46 | 6.218 | 4.483 | 5.680 | 6.237 | 4.511 | 5.598 | 2.741 | 2.832 |
| P3 | 12.028 | 7.068 | 8.884 | 1.563 | 0.787 | 1.092 | 1.548 | 0.779 | 1.086 | 3.840 | 3.720 |
| P4 | 7.304 | 5.467 | 5.900 | 3.062 | 2.596 | 2.800 | 3.072 | 2.568 | 2.790 | 4.466 | 4.614 |
| P5 | 6.800 | 5.300 | 5.800 | 7.592 | 3.779 | 6.459 | 7.657 | 3.745 | 6.462 | 3.465 | 3.403 |
| P6 | 11.232 | 3.660 | 9.801 | 19.757 | 6.415 | 17.435 | 19.562 | 6.331 | 17.669 | 2.511 | 2.488 |
| P7 | 3.424 | 2.261 | 2.894 | 0.040 | 0.024 | NA | 0.040 | 0.024 | NA | 3.439 | 3.519 |
| P8 | 16.518 | 12.078 | 14.454 | 1.116 | 0.481 | 0.589 | 1.101 | 0.487 | 0.585 | 2.858 | 2.829 |
| P9 | 5.871 | 4.054 | 4.700 | 2.915 | 2.612 | 2.604 | 2.919 | 2.587 | 2.640 | 2.560 | 2.563 |
| P10 | 5.085 | 3.782 | 4.557 | 8.395 | 3.644 | 6.191 | 8.420 | 3.690 | 6.114 | 4.309 | 4.133 |
| P11 | 4.300 | 3.200 | 3.800 | 3.200 | 2.600 | 2.800 | 3.210 | 2.650 | 2.820 | 4.089 | 4.160 |
| P12 | 3.294 | 3.135 | 3.200 | 2.100 | 1.600 | 1.200 | 2.150 | 1.560 | 1.650 | 4.555 | 4.556 |
| P13 | 1.353 | 0.777 | 1.100 | 1.800 | 1.400 | 1.100 | 1.820 | 1.560 | 1.600 | 3.200 | 3.147 |
| P14 | 5.200 | 3.800 | 4.200 | 2.800 | 2 | 2.600 | 2.823 | 1.982 | 2.550 | 3.100 | 3.162 |

Notes: (1) Lesion VOIs were semi-automatically delineated on real FDG PET/CT and transferred to the corresponding synthetic FDG PET images for quantitative measurement. One target lesion was selected for each patient. (2) NA indicates missing original data; SUVpeak was unavailable for P7 in both real and synthetic FDG images, and therefore the SUVpeak analysis included 13 cases. (3) Patient IDs P1–P14 correspond to those shown in Supplementary Figure S1. (4) Liver SUVmean was measured using ROIs placed at the same anatomical level on the real and synthetic FDG images. Abbreviations: OCT, ^18^F-octreotide; FDG, ^18^F-fluorodeoxyglucose; PET, positron emission tomography; SUV, standardized uptake value; NA, not available.

**Supplementary Table S2. Lesion-level agreement between real and synthetic ^18^F-FDG PET**

| **Parameter** | **n** | **Real FDG (mean ± SD)** | **Synthetic FDG (mean ± SD)** | **Pearson r** | **P value** | **Spearman ρ** | **Paired t-test P value** | **Bias (synthetic - real)** | **Difference SD** | **95% LoA lower** | **95% LoA upper** |
| --- | --- | --- | --- | --- | --- | --- | --- | --- | --- | --- | --- |
| SUVmax | 14 | 4.90±5.06 | 4.90±5.02 | 1 | <0.001 | 1 | 0.8157 | 0.004 | 0.0622 | -0.118 | 0.1259 |
| SUVmean | 14 | 2.61±1.75 | 2.61±1.74 | 0.9995 | <0.001 | 0.9945 | 0.7622 | 0.0047 | 0.0571 | -0.1072 | 0.1167 |
| SUVpeak | 13 | 4.41±4.50 | 4.48±4.49 | 0.9991 | <0.001 | 0.9986 | 0.2053 | 0.0728 | 0.1959 | -0.3112 | 0.4567 |
| Liver SUVmean | 14 | 3.40±0.75 | 3.39±0.75 | 0.9933 | <0.001 | 0.978 | 0.9669 | -0.001 | 0.0871 | -0.1717 | 0.1698 |

Notes: SUVpeak analysis included 13 cases because SUVpeak was unavailable for P7. Bias was calculated as synthetic FDG minus real FDG. The 95% limits of agreement were calculated as bias ± 1.96 SD of the paired differences. Abbreviations: FDG, ^18^F-fluorodeoxyglucose; PET, positron emission tomography; SUV, standardized uptake value; SD, standard deviation; LoA, limits of agreement.

**Supplementary Table S3. SSTR/FDG concordance classification in the test cohort**

| **Patient ID** | **OCT SUVmax** | **Real FDG SUVmax** | **Synthetic FDG SUVmax** | **Liver SUVmean** | **OCT/liver ratio** | **FDG/liver ratio** | **SSTR status** | **FDG status** | **Concordance class** |
| --- | --- | --- | --- | --- | --- | --- | --- | --- | --- |
| P1 | 3.607 | 8.042 | 8.098 | 2.405 | 1.500 | 3.344 | SSTR+ | FDG+ | Dual-positive (SSTR+/FDG+) |
| P2 | 61.226 | 6.218 | 6.237 | 2.741 | 22.334 | 2.268 | SSTR+ | FDG+ | Dual-positive (SSTR+/FDG+) |
| P3 | 12.028 | 1.563 | 1.548 | 3.840 | 3.132 | 0.407 | SSTR+ | FDG− | SSTR+/FDG− |
| P4 | 7.304 | 3.062 | 3.072 | 4.466 | 1.635 | 0.686 | SSTR+ | FDG− | SSTR+/FDG− |
| P5 | 6.800 | 7.592 | 7.657 | 3.465 | 1.962 | 2.191 | SSTR+ | FDG+ | Dual-positive (SSTR+/FDG+) |
| P6 | 11.232 | 19.757 | 19.562 | 2.511 | 4.474 | 7.870 | SSTR+ | FDG+ | Dual-positive (SSTR+/FDG+) |
| P7 | 3.424 | 0.040 | 0.040 | 3.439 | 0.996 | 0.012 | SSTR− | FDG− | Both-low (SSTR−/FDG−) |
| P8 | 16.518 | 1.116 | 1.101 | 2.858 | 5.778 | 0.391 | SSTR+ | FDG− | SSTR+/FDG− |
| P9 | 5.871 | 2.915 | 2.919 | 2.560 | 2.293 | 1.139 | SSTR+ | FDG+ | Dual-positive (SSTR+/FDG+) |
| P10 | 5.085 | 8.395 | 8.420 | 4.309 | 1.180 | 1.948 | SSTR+ | FDG+ | Dual-positive (SSTR+/FDG+) |
| P11 | 4.300 | 3.200 | 3.210 | 4.089 | 1.052 | 0.783 | SSTR+ | FDG− | SSTR+/FDG− |
| P12 | 3.294 | 2.100 | 2.150 | 4.555 | 0.723 | 0.461 | SSTR− | FDG− | Both-low (SSTR−/FDG−) |
| P13 | 1.353 | 1.800 | 1.820 | 3.200 | 0.423 | 0.562 | SSTR− | FDG− | Both-low (SSTR−/FDG−) |
| P14 | 5.200 | 2.800 | 2.823 | 3.100 | 1.678 | 0.903 | SSTR+ | FDG− | SSTR+/FDG− |

Notes: (1) A lesion-to-liver SUVmax ratio > 1.0 was used as the threshold for positive uptake. (2) Because OCT liver SUVmean data were unavailable, real FDG liver SUVmean was used as the common reference for both tracers; this represents a methodological limitation. (3) The OCT/liver ratio of P7 was 0.996 and was therefore classified as SSTR− according to the predefined threshold. No SSTR−/FDG+ lesion was identified in the current test cohort. Class distribution: SSTR+/FDG−, n = 5; SSTR−/FDG+, n = 0; SSTR+/FDG+, n = 6; SSTR−/FDG−, n = 3. Abbreviations: SSTR, somatostatin receptor; OCT, ^18^F-octreotide; FDG, ^18^F-fluorodeoxyglucose; SUV, standardized uptake value.

**Supplementary Figure S1. Four-quadrant distribution of SSTR and FDG uptake in the test cohort**

Supplementary Figure S1. Four-quadrant distribution of SSTR and FDG uptake in the test cohort. The x-axis represents the lesion FDG SUVmax-to-liver SUVmean ratio, and the y-axis represents the lesion OCT SUVmax-to-liver SUVmean ratio. Dashed lines indicate the threshold of 1.0 used for classification. Individual cases are labeled P1–P14. The distribution included 6 SSTR+/FDG+ cases, 5 SSTR+/FDG− cases, 3 SSTR−/FDG− cases, and 0 SSTR−/FDG+ cases.
