# Supplementary material for: Cascaded deep learning for synthesis of 18F-FDG PET from 18F-octreotide: model parameter optimization and performance evaluation
Source: Front Nucl Med. 2026 Oct 16;6:1920721. doi: 10.3389/fnume.2026.1920721 (PMC13719449; doi:10.3389/fnume.2026.1920721)
Supplement: Supplementary file 2 [file Supplementaryfile2.docx]

**Table S2.** Detailed training and inference configuration of the cascaded conditional GAN for both stages.

| Item | Stage 1 (3D U-Net GAN) | Stage 2 (Self-attention V-Net GAN) |
| --- | --- | --- |
| Framework | Pix2Pix conditional GAN | Pix2Pix conditional GAN |
| Generator | 3D U-Net | Self-attention V-Net 3D |
| Discriminator | Multi-scale 3D PatchGAN (full, 1/2, 1/4 resolutions) | Multi-scale 3D PatchGAN (full, 1/2, 1/4 resolutions) |
| Adversarial loss | LSGAN | LSGAN |
| Normalization | Instance normalization | Instance normalization |
| Weight initialization | Xavier initialization | Xavier initialization |
| Training epochs | 2000 | 500 |
| Batch size | 4 | 1 |
| Generator initial learning rate | 4 × 10⁻⁴ | 4 × 10⁻⁴ |
| Discriminator initial learning rate | 2 × 10⁻⁴ | 2 × 10⁻⁴ |
| λ_pix2pix (L1 weight) | 200 | 200 |
| λ_AB / λ_BA | 5.0 / 5.0 | 5.0 / 5.0 |
| Input crop size | 128 × 128 × 128 | 256 × 256 × 128 |
| Validation interval | Every 100 epochs | Every 100 epochs |
| Random seed | 10 | 10 |
| Inference strategy | Sliding window (batch size 1, overlap 0.5, Gaussian weighting) | Sliding window (batch size 1, overlap 0.5, Gaussian weighting) |
